# Estrutura das representares sociais da enfermagem sobre a morte decorrente da COVID-19*

**DOI:** 10.15649/cuidarte.2558

**Published:** 2023-05-28

**Authors:** Bruno Neves da Silva, Valéria Gomes-Fernandes-da-Silva, Erika Simone Galvao-Pinto, Nilba Lima-de-Souza, Francisco Arnoldo Nunes-de-Miranda

**Affiliations:** 1 . Universidade Federal do Rio Grande do Norte. Natal, RN, Brasil. Email: enfbneves@gmail.com Universidade Federal do Rio Grande do Norte Universidade Federal do Rio Grande do Norte Natal RN Brazil enfbneves@gmail.com; 2 . Universidade Federal do Rio Grande do Norte. Natal, RN, Brasil. Email: valeriafernandes7@hotmail.com Universidade Federal do Rio Grande do Norte Universidade Federal do Rio Grande do Norte Natal RN Brazil valeriafernandes7@hotmail.com; 3 . Universidade Federal do Rio Grande do Norte. Natal, RN, Brasil. Email: erikasg p@g mail.com Universidade Federal do Rio Grande do Norte Universidade Federal do Rio Grande do Norte Natal RN Brazil erikasg p@g mail.com; 4 . Universidade Federal do Rio Grande do Norte. Natal, RN, Brasil. Email: nilba.lima@hotmail.com Universidade Federal do Rio Grande do Norte Universidade Federal do Rio Grande do Norte Natal RN Brazil nilba.lima@hotmail.com; 5 . Universidade Federal do Rio Grande do Norte. Natal, RN, Brasil. Email: farnoldo@gmail.com Universidade Federal do Rio Grande do Norte Universidade Federal do Rio Grande do Norte Natal RN Brazil farnoldo@gmail.com

**Keywords:** COVID-19, Morte, Enfermagem, Pandemias, Pesquisa Qualitativa, COVID-19, Death, Nursing, Pandemics, Qualitative Research, COVID-19, Muerte, Enfermería, Pandemias, Investigación cualitativa

## Abstract

**Introdujo:**

Ainda que se saiba que a morte faz parte do ciclo da vida, diante de uma doenna com muitos aspectos ainda desconhecidos como a COVID-19, torna-se importante compreender como os trabalhadores de enfermagem representam esse fenómeno, uma vez que sao os únicos profissionais de saúde que continuam o cuidado ao indivíduo mesmo após a finitude da vida.

**Objetivo:**

analisar a estrutura das representanóes sociais sobre morte e morrer decorrentes de COVID-19 elaboradas por profissionais de enfermagem.

**Materiais e métodos:**

estudo qualitativo ancorado na vertente estrutural da Teoria das Representanóes Sociais com énfase na Teoria do Núcleo Central, desenvolvido junto a 32 profissionais de enfermagem da cidade de Natal, estado do Rio Grande do Norte, Brasil. A coleta de dados foi realizada online mediante utilizanao da técnica de associanao livre de palavras. Para a análise dos dados recorreu-se as análises prototípica e de similitude.

**Resultados:**

o provável núcleo central das representanóes foi constituído pelos termos tristeza e medo, e a composinao do sistema periférico e da zona de contraste reforgam esse núcleo.

**Discussao:**

o núcleo central das representanóes dos profissionais de enfermagem sobre morte e morrer decorrentes de COVID-19 se constitui em um Themata polémico de representanao, e destaca os prejuízos a saúde psicossocial dos trabalhadores de enfermagem da linha de frente.

**Conclusoes:**

perante as representares elaboradas sobre a morte e morrer decorrentes de COVID-19, é necessário o desenvolvimento de estratégias de enfrentamento que colaborem para a saúde psicossocial dos trabalhadores de enfermagem.

## Abstract (en)

**Introduction:**

Although it is known that death is part of the life cycle, in the face of a disease with many aspects still unknown, such as COVID-19, it is important to understand how nursing workers represent this phenomenon, since they are the only professionals that continue to care for the individual even after the end of life.

**Objective:**

to analyze the structure of social representations about death and dying due to COVID-19 elaborated by nursing professionals.

**Materials and methods:**

qualitative study anchored in the structural aspect of the Theory of Social Representations with emphasis on the Theory of the Central Nucleus, developed with 32 nursing professionals from the city of Natal, state of Rio Grande do Norte, Brazil. Data collection was performed online using the free word association technique. For data analysis, prototypical and similarity analyzes were used. Results: the probable central nucleus of the representations was constituted by the terms sadness and fear, and the composition of the peripheral system and the contrast zone reinforce this nucleus.

**Discussion:**

the core of nursing professionals' representations about death and dying from COVID-19 constitutes a controversial issue of representation and highlights the damage to the psychosocial health of frontline nursing workers.

**Conclusions:**

in view of the elaborated representations about death and dying resulting from COVID-19, it is necessary to develop coping strategies that contribute to the psychosocial health of nursing workers.

## Resumen

**Introducción:**

Si bien se sabe que la muerte es parte del ciclo de vida, ante una enfermedad con muchos aspectos aún desconocidos, como lo es el COVID-19, es importante comprender cómo los trabajadores de enfermería representan este fenómeno, ya que son los únicos profesionales que continuan cuidando al individuo incluso después del final de la vida.

**Objetivo:**

analizar la estructura de las representaciones sociales sobre la muerte y el morir por COVID-19 elaboradas por profesionales de enfermería.

**Materiales y métodos:**

estudio cualitativo anclado en el aspecto estructural de la Teoría de las Representaciones Sociales con énfasis en la Teoría del Núcleo Central, desarrollado con 32 profesionales de enfermería de la ciudad de Natal, estado de Rio Grande do Norte, Brasil. La recolección de datos se realizó en línea utilizando la técnica de asociación libre de palabras. Para el análisis de los datos se utilizaron análisis prototípicos y de similitud. Resultados: el probable núcleo central de las representaciones estuvo constituido por los términos tristeza y miedo, y la composición del sistema periférico y la zona de contraste refuerzan este núcleo.

**Discusión:**

el núcleo de las representaciones de los profesionales de enfermería sobre la muerte y el morir por COVID-19 constituye un tema de representación controvertido, y destaca el daño a la salud psicosocial de los trabajadores de enfermería de primera línea.

**Conclusiones:**

frente a las representaciones elaboradas sobre la muerte y el morir como consecuencia de la COVID-19, es necesario desarrollar estrategias de enfrentamiento que contribuyan a la salud psicosocial de los trabajadores de enfermería.

## Introdujo

A abordagem sobre a morte demanda conhecer as maneiras que as pessoas lidam e interpretam essa fase do ciclo da vida, a qual perpassa o cotidiano das pessoas. A relagáo com a morte se modificou durante o transcorrer da história. Logo, a compreensáo sobre a morte náo se refere apenas a finitude da vida biológica, mas leva em considerares aspectos simbólicos, culturais e históricos, socialmente elaborados. No atual contexto da COVID-19, a presenta da morte, já cercada de dificuldades, adquire um caráter mais penoso para todos os indivíduos implicados no processo: trabalhadores da saúde, pacientes e seus entes queridos^1^.

Lidar com situagóes de morte e morrer em larga escala, como ocorrido durante a pandemia da COVID-19, acarreta prejuízos a saúde psicossocial dos profissionais de enfermagem, o que demanda o desenvolvimento de estratégias significativas de preparo para esses profissionais em suas práticas de cuidado frente a morte, por sua peculiaridade, como os únicos profissionais de saúde a cuidarem do indivíduo após a finitude da vida^2^^,^^3^. Por caracterizar-se como uma infecgáo viral aguda, letal e altamente transmissível, que tem ocasionado impactos repercutidos globalmente, a pandemia da COVID-19 despertou uma maior preocupagáo com a saúde mental dos indivíduos^4^^,^^5^.

Nesse cenário, discussóes sobre a saúde mental dos trabalhadores da saúde, que assistem os pacientes diretamente acometidos pela doenga e considerados como “linha de frente" na assisténcia, tornaram-se evidentes e necessárias, diante da soma de fatores que circundam a rotina de trabalho. Esse contexto náo difere para os trabalhadores de enfermagem, que, além da crise, tém de lidar com jornadas de trabalho extenuantes, baixa remuneragáo, dentre outros fatores que interferem na saúde psicossocial^2^.

É nessa perspectiva global que Macedo^6^, pautando-se no referencial de Elisabeth Klüber-Ross sobre a morte e o processo de morrer^7^, defende a inclusáo dessa temática no discurso social e nas práticas educativas, visando tornar o processo menos penoso para aqueles implicados nele (incluindo os profissionais de saúde), e humanizar as práticas de cuidado frente a terminalidade, uma vez que a morte continua representada como um tabu, sobretudo nas sociedades ocidentais.

Ainda que se saiba que a morte faz parte do ciclo da vida, diante de um vírus novo e uma doenga com muitos aspectos ainda desconhecidos, torna-se importante compreender como os profissionais se sentem preparados para o seu enfrentamento, especialmente os profissionais de enfermagem, cujo exercício profissional requer maior, em comparagáo aos demais profissionais, tempo de permanéncia junto aos pacientes^3^. Ressalta-se que as crengas sobre a morte sáo influenciadas pelo meio sociocultural que, conscientemente ou náo, moldam emogóes, sentimentos e condutas, maximizadas em situagóes de crise a medida que os profissionais da saúde tém de lidar com o sofrimento físico, social, emocional e espiritual perante a morte do outro, o que lhes remete ao reflexo da sua própria morte, e vem a causar danos psicossociais^8^.

Dessa forma, as representagóes sociais sobre a morte e o morrer náo sáo resultantes apenas da finitude da vida biológica, constituindo-se em um processo de interpretagáo construído e partilhado em diferentes contextos históricos e socioculturais, e, assim como outros fenómenos da vida social, as diversas leituras sobre a morte e o morrer tem determinado distintas interpretagóes e influenciado as formas de enfrentamento, e a assisténcia ao indivíduo moribundo^9^.

Ante ao exposto, compreender como os profissionais de enfermagem representam o processo de morte e morrer decorrentes da COVID-19 pode contribuir para desenvolver estratégias efetivas de cuidado voltadas para esses profissionais, que podem ser caracterizadas como um tipo de prevengáo quinquenária, ou seja, com foco no cuidador, do qual, efetivamente, emergem todos os cuidados^10^. Ademais, a compreensáo dessas representares oportuniza refletir sobre as formas de cuidado elaboradas pela enfermagem, possibilitando aprimorar esse cuidado a partir das perspectivas dos profissionais implicados nele.

O presente estudo objetivou, dessa forma, analisar a estrutura das representagóes sociais sobre a morte e o morrer decorrentes de COVID-19 elaboradas por profissionais de enfermagem no transcurso da pandemia ocasionada pela doenga. Partiu-se do questionamento norteador: qual a estrutura das representagóes sociais de profissionais de enfermagem sobre a morte e o processo de morrer em decorréncia da COVID-19?

## Materiais e Método

Estudo qualitativo ancorado na vertente estrutural do referencial teórico-metodológico da Teoria das Representares Sociais (TRS), com énfase na Teoria do Núcleo Central (TNC). Para Abric^11^^,^^12^, toda representado se encontra organizada ao redor de um núcleo central que determina concomitantemente a sua significado e organizado internas, núcleo este que se constitui em um subconjunto da representado, sendo composto por elementos que, caso ausentes, desestruturariam a representado ou atribuiriam a ela um significado totalmente distinto.

Intencionalmente, em fundo da pandemia, o estudo foi desenvolvido online no período de margo a abril de 2021, com profissionais de saúde responsáveis pelos cuidados prestados aos pacientes acometidos pelos casos de COVID-19 no município de Natal, localizado no Estado do Rio Grande do Norte, na regiáo Nordeste do Brasil. Optou-se em náo estabelecer o universo dos participantes a priori, respeitando-se as condigóes elegíveis para participar da pesquisa: profissionais de enfermagem maiores de 18 anos, em exercício profissional na linha de frente, prestando assisténcia direta aos pacientes acometidos pela COVID-19, e que aceitassem, de forma espontanea e consentida, fazer parte do estudo. Destaca se, contudo, que foi estabelecido um número mínimo de 25 participantes a ser atingido, conforme recomendado por alguns autores^13^. Cumpre-se dizer que o número de participantes náo se constitui no fato mais relevante, mas sim, a constatagáo de que o objeto de pesquisa seja motivo de pertencimento e atravessamento para os participantes do estudo, característica observada no estudo em tela e já relatada na literatura^14^.

Definiu-se para a produgáo dos dados a criagáo de um instrumento para aplicagáo da técnica de associagáo livre de palavras (TALP), a qual favorece a revelagáo de desejos e elementos fundamentais na apreensáo de modo fidedigno dos significados atribuídos a um estímulo sobre uma determinada pesquisa, possibilitando uma melhor compreensáo do objeto e fenómeno a se investigar, permitindo, ainda, evidenciar campos semanticos por meio dos termos comuns levantados pelos sujeitos a partir de uma indugáo^15^. Utilizou-se como indutor a seguinte pergunta: “quais sáo as primeiras cinco palavras que vém a sua mente quando eu lhe digo morte por COVID-19?". Foram coletadas, ainda, algumas informagóes sociodemográficas para caracterizagáo dos participantes.

Operacionalmente, utilizou-se a ferramenta Chat-Forms®, que permitiu a aplicagáo do instrumento utilizado (criado mediante utilizagáo da plataforma Google Forms®), simulando um chat de mensagens entre os participantes da pesquisa e a tecnologia. Assim, gerou-se um link, formulando um convite de participado, juntamente com o Termo de Consentimento Livre e Esclarecido (TCLE). O envio para os participantes profissionais de enfermagem se deu por meio de redes sociais (WhatsApp® e Instagram®).

Findada a fase de coleta de dados, encerrada após um período de duas semanas sem nenhuma resposta nova aos formulários, os dados obtidos foram organizados em planilhas, e o conteúdo da TALP em uma matriz no software LibreOffice®. Procedeu-se a lematizagáo das evocagóes, que consiste na redugáo das palavras ao mesmo radical; e a sua categorizagáo, agrupando as palavras que se assemelharam em seus sentidos e significados, com o objetivo de evitar ambiguidades. Posteriormente, exportou- se a matriz para análise prototípica e de similitude pelo software Interface de R pour les Analyses Multidimensionnelles de Textes et de Questionnaires (IRAMUTEQ®). A matriz de dados foi armazenada na plataforma Zenodo^16^.

A análise prototípica analisa as saliéncias dos elementos de representado mediante a frequéncia que a evocagáo é feita e a ordem que o termo ou expressáo aparece, organizando os resultados em um quadro de quatro casas, que representa um plano cartesiano no qual o núcleo central da representagáo social se encontra localizado no quadrante superior esquerdo (QSE), sendo composto por palavras evocadas por maior número de indivíduos e nas primeiras posigóes, ou seja, prontamente evocadas^11^^,^^17^.

O conteúdo de cada quadrante do quadro de quatro casas, fornece informagóes relevantes sobre as representagóes sociais. O quadrante superior direito (QSD) representa a primeira periferia, que abrange respostas com saliéncia, porém considerados secundários da representagáo. A segunda periferia, por sua vez, localiza-se no quadrante inferior direito (QID), e inclui as evocagóes pouco salientes, e menos interessantes para a estrutura da representagáo do grupo, indicando aspectos mais particularizados. A zona de contraste ocupa o quadrante inferior esquerdo (QIE), cujas evocagóes podem representar uma complementagáo da primeira periferia, ou a existéncia de um subgrupo que valoriza elementos diferentes da maioria, por terem frequéncia baixa e menor ordem média de evocagáo (OME)^11^^,^^17^.

A constituigáo do quadro de quatro casas pelo IRAMUTEQ®, calcula a frequéncia de ocorréncia das palavras evocadas e sua OME, que representa a posigáo média em que o termo apareceu na classificagáo conforme a ordem das evocagóes. Numericamente, atribui-se valores de um a cinco as respostas (primeira palavra evocada recebe valor um, e assim sucessivamente), possibilitando visualizar quais palavras foram mais prontamente evocadas (com maior rapidez)^13^. Tem-se no QSE palavras com alta frequéncia e baixa OME, no QSD, palavras com alta frequéncia e alta OME, no QIE palavras com baixa frequéncia e OME, e no QID, evocagóes com baixa frequéncia e alta OME.

A análise de similitude, por sua vez, baseia-se na teoria dos grafos e permite visualizar as conexóes entre as palavras evocadas e suas relagóes intra e entre os quadrantes do quadro de quatro casas (mais fortes conforme mais grossas as arestas que as interligam)^18^. Recorreu-se a essa análise para auxiliar a estabelecer a centralidade dos elementos, devido ao fato de que nem todo elemento apontado no QSE do quadro de quatro casas constituir-se em um elemento central da representagáo, podendo elementos presentes nos demais quadrantes representarem elementos centrais^13^.

O resultado do processamento da análise prototípica e de similitude pelo IRAMUTEQ® foi apresentado mediante utilizagáo gráfica e de um quadro. A análise da estrutura das representagóes foi interpretada a luz da TRS com énfase na TNC. Recorreu-se, ainda, as reflexóes elaboradas por Kübler-Ross^7^ sobre o processo de morte e morrer.

Quanto aos preceitos éticos, este estudo obedeceu a todos os pressupostos da Resolugáo 510/2016 do Conselho Nacional de Saúde^19^ e a Lei n° 13.709/2018 (Lei Geral de Protegáo de Dados) do Brasil.

Ressalta-se que os individuos que participaram do estudo nao foram passíveis de identificado pelos pesquisadores por nenhum meio.

## Resultados

Participaram do estudo 32 profissionais de enfermagem, sendo 81,25% (n = 26) enfermeiros, e 18,75% (n = 6) técnicos em enfermagem. Quanto ao sexo, 93,75% (n = 30) declararam-se do sexo feminino, e 6,25% (n = 3), do sexo masculino. A média de idade foi de 32,83 anos, com valor mínimo de 23 e máximo de 53 anos. O tempo médio de formado dos profissionais foi de 7,16 anos. O tempo de resposta do formulário foi apontado por todos (100%) os participantes da pesquisa como entre um e cinco minutos.

Quanto a análise prototípica, foram constatadas 160 (100%) evocagóes, sendo 32 (20%) diferentes entre si, indicando homogeneidade do corpus. Adotou-se tres como a frequéncia mínima, e, após a exclusao das evocagóes com frequéncia inferior a esse número, obteve-se um aproveitamento de 113 termos evocados (70,6%). A frequéncia média obtida e utilizada para a construgao do quadro de quatro casas foi de 5,38, e a OME foi 2,81 em um ranking de 1 a 5. A distribuigao das evocagóes em cada quadrante do quadro de quatro casas viabilizou a análise da estrutura da representagao, que pode ser visualizada no Quadro 1.

**Quadro 1:** Quadro de quatro casas resultante da análise prototípica das evocagóes. Natal, RN, Brasil, 2021.

OME < 2,81				OME > 2,81		
	NÚCLEO CENTRAL (QSE)			PRIMEIRA PERIFERIA (QSD)		
		f	OME		f	OME
f > 5,38	Medo	17	2,1	Sofrimento	7	3
	Tristeza	15	2	Família	6	3,8
	Dor	7	2,4	Desespero	6	3,5
	Impotencia	6	2,5	Angústia	6	3,5
	ZONA DE CONTRASTE (QIE)			SEGUNDA PERIFERIA (QID)		
		f	OME		f	OME
f > 5,38	Solidao	4	1,2	Prevendo	4	3
	Isolamento	3	2,7	Agonia	4	3
	Perda	3	2,7	Alta_incidencia	4	4
	Superlotado_hospitalar	3	2	Política	3	4,3
				Saudade	3	4
				Vacina	3	3
				Desanimo	3	4.3
				Ansiedade	3	3,7

No QSE, apareceram as palavras “medo”, “tristeza”, “dor” e “impoténcia”, as quais foram evocadas prontamente por um número considerável de participantes do estudo, o que destaca que essaspalavras podem representar o núcleo central das representagóes sociais dos profissionais de enfermagem sobre o processo de morte e morrer decorrentes da COVID-19.

**Figura 1:**
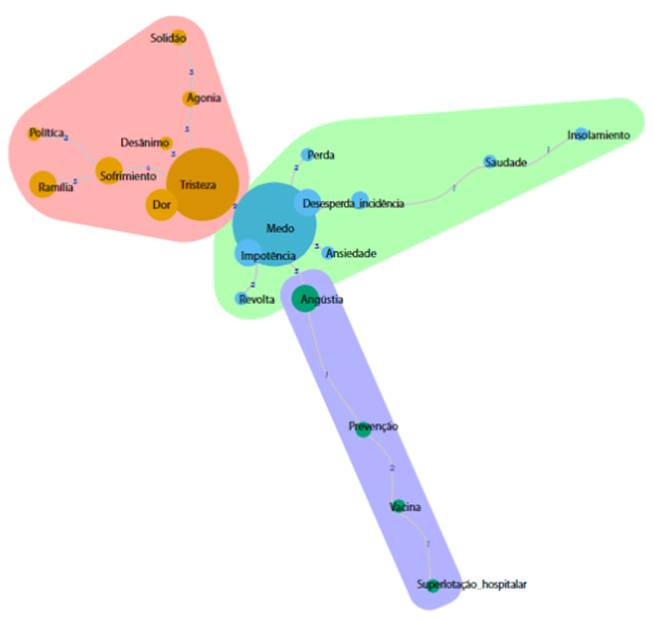
Representagóes

No entanto, observou-se evocagóes com frequéncia semelhante, ainda que com OME diferentes, o que pode denotar que algumas palavras que figuraram no núcleo central podem estar transitando, na verdade, na periferia da representado, remetendo a um transito de saberes sobre morte e morrer na pandemia. Para melhor visualizar esse pressuposto, explorou-se a análise de similitude representada na Figura 1, para estabelecer a centralidade dos elementos.

Analisando a Figura 1, reflete-se que o núcleo central das representagóes pode ser representado pelas evocagóes “medo” e “tristeza”, e que os termos “dor” e “impoténcia” se posicionam na periferia desse núcleo, juntamente com “sofrimento”, ” família”, “desespero” e “angústia”.

Para mais, observa-se que as evocagóes que aparecem na segunda periferia se encontram mais afastadas do núcleo central, com excegao de "alta incidencia", o que denota que a alta incidencia de processos de morte e morrer relacionados a COVID-19 ocasiona medo e tristeza nos profissionais de enfermagem, podendo essa evocagao estar mais intimamente próxima do sistema periférico de representagao do que as demais palavras do QID do quadro de quatro casas.

Os termo "perda" evocado na zona de contraste, dada a conexao estabelecidas na análise de similitude, e o fato de nao se encontrar tao afastado quanto as demais evocagóes dessa zona, presume-se que seja complementar a primeira periferia, enquanto as evocagóes "superlotagao hospitalar", "isolamento" e "solidao", apesar de seu conteúdo estar fortemente relacionado ao processo de morte e morrer pela doenga, permaneceram mais afastados, podendo representar a existencia de subgrupos de profissionais que valorizam esses termos mais do que outros.

## Díscussao

O provável núcleo central das representares sociais da equipe de enfermagem sobre morte e morrer decorrentes da infecgáo pelo novo coronavirus é constituido pelos termos tristeza e medo, que possuem a fungáo de gerar, organizar e estabilizar a representado social sobre esse assunto. Destaca se que esse núcleo pode representar a tristeza dos profissionais frente ao processo de morte pela COVID-19, que foi vivenciado de forma intensa, assim como o medo que os profissionais manifestam de vivenciá-lo^12^^,^^20^.

O provável núcleo central constatado encontra suporte em outros estudos da literatura, que destacam, por exemplo, que a enfermagem vivencia um sofrimento intenso durante o acompanhamento do processo de morte e morrer dos pacientes, diferentemente do que se difunde, de que os trabalhadores se acostumariam com a ocorrencia da morte^21^.

Em estudo desenvolvido com 178 enfermeiros no estado do Ceará, Brasil, pesquisadores analisaram a estrutura das representares sociais desses profissionais sobre a COVID-19, e encontraram que o termo medo figurava em um possivel núcleo central dessas representares, e o termo tristeza foi evidenciado como localizado na segunda periferia. Ainda que o estudo náo tenha tratado especificamente sobre a morte e o morrer, a evocado "morte" apareceu na primeira periferia das representares elaboradas pelos profissionais, dando suporte ao provável núcleo central^22^.

Destaca-se que a transformado das representares sociais náo ocorre, exclusivamente, por palavras ou informares (pouco impacto), mas por ares, novas práticas, ou seja, uma transformado brutal ou abrupta através de eventos ou atos rápidos, imediatos, determinados por um evento especifico que envolve um elemento central, percebido como irreversivel, ou seja, uma situado de crise^12^, a exemplo da que se circunscreve na pandemia da COVID-19.

Para que uma representado social se transforme verdadeiramente, deve haver uma transformado de seu núcleo central. Nesse sentido, o núcleo central, possui, de um lado, a fundo geradora, de dar a representado seu significado; do outro, a fundo de organizado, determinando a natureza das ligares entre os elementos, o que confere as representares sociais uma propriedade estável^11^^,^^12^.

Os prováveis componentes do núcleo central do presente estudo foram destacados como sentimentos em um estudo transversal realizado no Brasil com 979 profissionais de saúde, em sua maioria enfermeiros e técnicos de enfermagem, em que medo, ansiedade e tristeza foram os sentimentos mais comuns descritos pelos participantes, sobretudo pela equipe de enfermagem, que passa maior parte do tempo junto aos pacientes^23^. O risco de exposigáo ao virus, insuficiencia de insumos e equipamentos necessários para desenvolver o cuidado, assim como o acesso limitado a servidos de saúde para abordagem do sofrimento psicológico foram fatores relacionados ao desenvolvimento desses sentimentos^24^.

O sistema periférico das representares sociais, por sua vez, tem a fundo de realizado e regulado do núcleo central, o que permite a modulado personalizada e a fundo de defesa, como um sistema que protege o núcleo central. Permite, ainda, a existencia de conotares e histórias individuais, sendo flexivel e tolerável a contradices e a heterogeneidade do grupo, contrastando com o núcleo central, o qual define a homogeneidade do grupo, e está ligado a sua memória, sendo consensual, rígido, e resistente a mudanzas^11^^,^^12^.

Nessa perspectiva, apreendeu-se que os termos evocados no sistema periférico do presente estudo podem incorporar representares de aspectos mais heterogéneos entre os participantes. As evocagóes "sofrimento", "desespero", "angústia" e "dor", de maneira geral, podem relacionar-se aos sentimentos aflorados em um contexto pandémico novo e desconhecido, largamente midiatizado, e com sérias repercussóes e mortalidade global, sendo esperado que esses sentimentos venham a tona nos trabalhadores de enfermagem diretamente implicados no cuidado aos indivíduos acometidos, e sendo os únicos profissionais a cuidarem do indivíduo mesmo após a finitude da vida. O termo família, por sua vez, pode destacar o medo (que figura no núcleo central) dos profissionais em perder seus entes queridos para a doenga, ou mesmo o receio de que eles próprios possam ser uma fonte de infecgáo. Tal receio é considerado, inclusive, um entrave no processo de trabalho^2^.

No que concerne aos termos observados na segunda periferia, apesar de indicarem, conforme Abric^11^, aspectos mais particularizados da representado, evocagóes como "ansiedade" e "desanimo" também apareceram como sentimentos ou representagóes centrais relacionadas a pandemia, e depreende-se dos termos "prevengáo", "política", "vacina" e "alta incidéncia", uma articulagáo relacionada ao contexto político brasileiro (além da relagáo intrínseca entre as evocagóes "vacina" e "prevengáo"), em que a náo adogáo de medidas mais duras de combate a pandemia, juntamente com a inércia do governo em incentivar medidas preventivas, assim como a veiculagáo de notícias falsas, colaboraram para a alta incidéncia evocada da doenga em território nacional^25^.

As representagóes sociais dos resultados obtidos pela análise prototípica e de similitude, nessa perspectiva, remetem a uma condigáo de transicionalidade entre os domínios do quadro de quatro casas e seus termos fundantes, em que se pode afirmar, tratar-se de representagóes polémicas^26^. Sáo efeitos previsíveis de mecanismos inconscientes sobre morte e morrer na pandemia pela COVID-19 as representagóes polémicas que provém justamente de contextos conflituosos e de outros aspectos circunstanciais, incluindo uma identidade social. Nesse sentido, a pandemia da COVID-19 e seu potencial de morte e morrer, confere uma certa identidade, embora negada ou pouco aclarada.

Kluber-Ross^7^ sobre o temor da morte, destacou cinco estágios (negagáo e isolamento, raiva, barganha, depressáo e aceitagáo), que, contudo, náo estáo claramente definidos no cenário do estudo, reconceituados pelos profissionais da pesquisa, sugerindo uma compreensáo como algo randomizado, transitando entre o núcleo central, as duas periferias e a zona de contraste, embora interligados. Assim, do ponto de vista estrutural, as representagóes sociais polémicas e na concepgáo linguística podem ser entendidas como Themata (Tema).

Sobre Themata, configura-se como um paradoxo, analisando-se a passagem de um nível microssocial para um nível macrossocial. Essa constatagáo está apoiada na concepgáo estrutural das representagóes sociais e na TNC, introduzindo na TRS, o conceito de Themata, que emerge da dificuldade em encontrar uma boa articulagáo entre os níveis micro e macro de análise. Em si mesmas, as thematas residem no senso comum, e náo na ciéncia, sáo antinomias oposicionais no processo do pensamento, exemplificados nuclearmente, através do medo. Medo que remete a condigáo mais instintiva do ser humano^26^.

O "medo", do ponto de vista conceitual de Themata, presente no núcleo central é reforgado estruturalmente na zona de contraste pela "solidáo" (depreende-se, relacionada a hospitalizagáo e afastamento dos pacientes de seus entes queridos, reforgado pela impossibilidade de visitas), "isolamento" (seja da própria familia, de lagos afetivos, de amizades, para protege-los do virus), e "perda" (relacionada a própria morte). Esse "medo", como Themata, é organizado com o apoio dos termos que remetem ao corpo físico, emogóes e sentimentos, tais como: "tristeza", "dor", "agonia" e "impotencia".

Prosseguindo na perspectiva conceitual de Themata, sobre o "medo", as duas periferias reforgam-no pelo "sofrimento", "desespero", "angústia", "agonia", "saudade", "desanimo", e "ansiedade", nao menos significativos pelos aspectos processuais da análise prototipica e de similitude, atribuindo um sentido voltado para a "familia", "prevengao", "alta incidencia", "politica" e "vacina".

Do ponto de vista conceitual do processo morte e o morrer demonstrado em Kluber-Ross^7^, o evento pandemico pela COVID-19 ocasiona esse movimento inconsciente entre os cinco estágios estabelecidos pela autora, nao estando claramente definidos, por se tratar de thematas e ressignificados com linguajar mais contemporaneo, até sob a influencia da exposigao e bombardeio midiático da pandemia da COVID-19. Supóe-se o processo de morte e morrer como um constante processo do pensamento, derivando emogóes, sentimentos e atitudes, organizado em termos de processos orientados na diregao de "Temas" comuns, tomados como a origem daquilo a que nos referimos cada vez, como conhecimento aceito ou mesmo como ideias primárias^26^.

Do ponto de vista da concepgao linguistica através de Themata, as representagóes sociais apresentam na concepgao moscoviciana tres dimensóes que conferem um panorama ou dimensao e do sentido que encerra: informagóes, campo representacional e atitude. O primeiro remete aos conceitos sob o dominio de um determinado grupo social. O segundo, dá a ideia de imagem, de modelo social ao conteúdo de um aspecto preciso da representagao. O terceiro, diz respeito a orientagao global em relagao ao objeto da representagao social^26^.

O conhecimento sobre as atitudes dos profissionais de enfermagem perante a morte no contexto da COVID-19 possibilita analisar uma parcela dos impactos ocasionados pelo problema no preparo desses trabalhadores para o enfrentamento desse tipo de fenómeno, permitindo, ainda, apontar suas atitudes no contexto da finitude dos pacientes, aspecto que recebe pouco enfoque na formagao e qualificagao profissional^3^.

Ante a análise estrutural das representagóes, cumpre-se reforgar a necessidade de intervengóes no sentido de fortalecer a saúde psicossocial dos trabalhadores de enfermagem, envolvendo a instituigao de saúde (que deve garantir suporte), e toda a equipe, que valorizem o apoio entre os profissionais e englobem desde a prevengao e a promogao da saúde mental, até a garantia do tratamento e a reabilitagao dos profissionais. Para mais, é mandatório a garantia de condigóes de trabalho, recursos e insumos necessários para que esses profissionais sejam capazes de oferecer uma assistencia segura para os individuos e para si próprios^27^.

Intervengóes como o apoio psicológico especializado, teleconsulta com escuta ativa, realizagao de práticas integrativas e complementares, exercicios de relaxamento e oferecimento de servigos gratuitos de saúde mental podem contribuir efetivamente na saúde biopsicossocial desses profissionais^28^.

Destaca-se, como limitagóes do presente estudo, o quantitativo restrito de profissionais participantes, o que aponta para a dificuldade de adesao de profissionais de enfermagem a pesquisas cientificas na modalidade remota. Contudo, reforga-se que o desenvolvimento de estudos sobre as representagóes desses profissionais sobre a COVID-19 possibilita subsidiar a proposigao de tecnologias cuidativo- educacionais que qualifiquem sua atuagao no cuidado aos individuos acometidos pela doenga^14^.

## Conclusoes

A análise estrutural das representajóes sociais de profissionais de enfermagem sobre o processo de morte e morrer decorrentes de COVID-19 destaca um núcleo central provavelmente constituído pelos termos medo e tristeza, que sao reforjados pelas evocajóes localizadas na zona de contraste e no sistema periférico de representado desses profissionais sobre o assunto, que se constitui em um Themata polémico, considerando os pressupostos de Moscovici, precursor da TRS.

Considerando essas representajóes, pode-se inferir que vivenciar o processo de morte e morrer decorrentes da COVID-19 ocasiona sérias repercussóes para a saúde psicossocial dos trabalhadores de enfermagem. Nessa perspectiva, cabe aos servijos de saúde elaborar intervenjóes de prevenjao quinquenária capazes de desenvolver estratégias de enfrentamento efetivas, assim como o oferecimento de apoio psicossocial, que auxiliem os profissionais a desenvolver atitudes de enfrentamento no sentido de frear o desgaste ocasionado pelo processo de trabalho na linha de frente contra o vírus e a pandemia.
